# P-1504. Propensity-Matched Comparison of Early vs. Late Antibiotic Therapy in *Stenotrophomonas maltophilia* Pneumonia: A Five-Hospital Study

**DOI:** 10.1093/ofid/ofae631.1673

**Published:** 2025-01-29

**Authors:** Elizabeth May, Ashlan Kunz Coyne

**Affiliations:** University of Kentucky College of Pharmacy, Lexington, Kentucky; University of Kentucky College of Pharmacy, Lexington, Kentucky

## Abstract

**Background:**

*Stenotrophomonas maltophilia* is a leading drug-resistant nosocomial pathogen worldwide. First-line antibiotic therapy for *S. maltophilia* is not a routine part of empiric antibiotic coverage. This study aimed to evaluate clinical outcomes of patients receiving early versus late antibiotic therapy for *S. maltophilia* pneumonia (PNA).

**Table 1.**
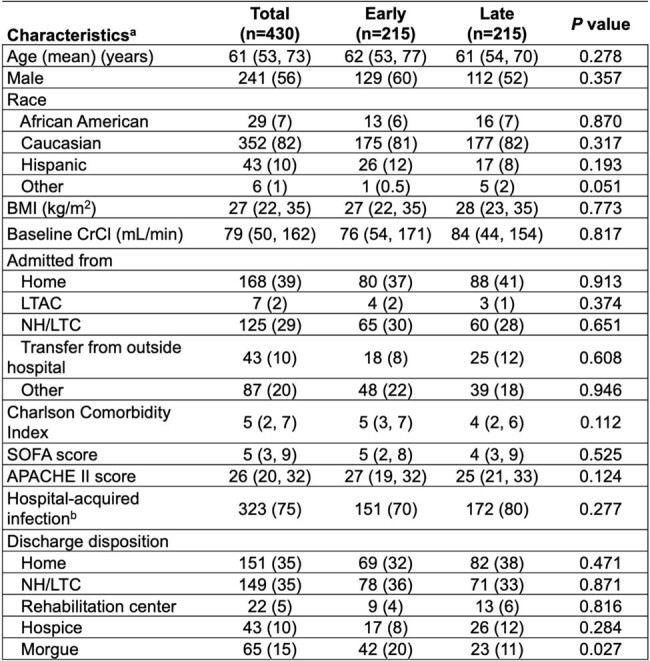
Baseline and Clinical Course Characteristics

aData are presented as “number (%)” or “median (interquartile range)”, as appropriate.

Abbreviations: BMI, body mass index; CrCL, creatinine clearance; LTAC, long-term acute care; NH/LTC, nursing home/long-term care.

**Methods:**

A retrospective, observational study was conducted at five hospitals within University of Kentucky HealthCare. Patients treated for *S. maltophilia* PNA between 1/1/2014 and 12/31/2023 receiving sulfamethoxazole/trimethoprim, minocycline or levofloxacin with reported in-vitro susceptibility were included. Patient and bacterial characteristics, all-cause mortality, and desirability of outcome ranking (DOOR) at 30 days were compared between patients receiving early (≤48 hours of culture collection) versus late ( >48 hours of culture collection) therapy (Table 1). Propensity-score matching (PSM) was used to adjust confounding. Subgroup and sensitivity analyses were used to verify the robustness of results.

**Table 2.**
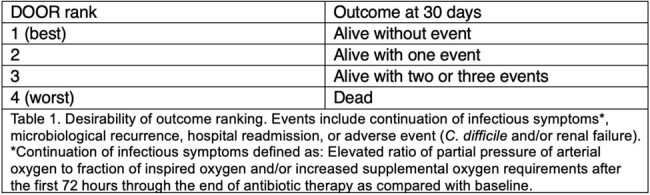
Desirability of Outcome Ranking (DOOR)

**Results:**

Median age of patients was 64 years (interquartile range [IQR] 52-71 years). The median Charlson Comorbidity Index and SOFA score were 4 (IQR 2-6) and 4 (2-7). At the time of index culture collection, 219/430 (50.9%) patients were in the intensive care unit, and 142/430 (33%) patients were on mechanical ventilation. Overall mortality at 30 days was 103/430 (24%). DOOR distribution of outcomes is shown in Figure 1. A randomly selected patient with early antibiotic administration had a 69% (95% CI 56%-79%, p< 0.001) likelihood of a better outcome as compared to a randomly selected patient receiving late antibiotic therapy.

**Figure 1.**
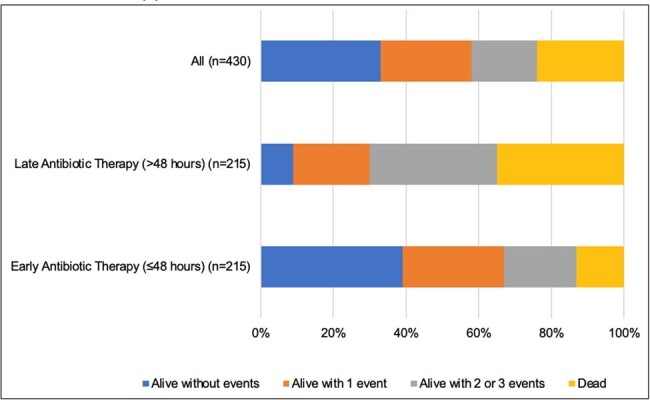
DOOR Analysis of All Patients with S. maltophilia Pneumonia Receiving Early vs. Late Antibiotic Therapy

**Conclusion:**

*S. maltophilia* PNA is associated with poor outcomes, especially in patients receiving therapy with in-vitro activity >48 hours from index culture collection.

**Disclosures:**

**All Authors**: No reported disclosures

